# A prospective, observational cohort study of patients presenting to an emergency department with acute shoulder trauma: the Manchester emergency shoulder (MESH) project

**DOI:** 10.1186/s12873-017-0149-y

**Published:** 2017-12-22

**Authors:** Michael J. Callaghan, Janos P. Baombe, Dan Horner, Charles E. Hutchinson, Dilraj Sandher, Simon Carley

**Affiliations:** 1 Emergency Department, Manchester University NHS Foundation Trust, Oxford Road, Manchester, M13 9WL UK; 2 Department of Health Professions, Brooks Building, Birley Campus, Manchester Metropolitan University, Manchester, M15 6GX UK; 3 Emergency Department, Salford Royal NHS Foundation Trust, Stott Lane, Salford, M6 8HD UK; 4 Warwick Medical School, Population Evidence and Technologies, University of Warwick, Coventry, CV4 7AL UK; 5 Department of Orthopaedic Surgery, Stepping Hill NHS Foundation Trust, Poplar Grove, Hazel Grove, Stockport, SK2 7JE UK; 6 Department of Radiology, University Hospitals Coventry and Warwickshire, Clifford Bridge Road, Coventry, CV2 2DX UK

**Keywords:** Emergency medicine, Magnetic resonance imaging, Shoulder, Trauma, Soft tissue injury

## Abstract

**Background:**

Fracture and dislocation of the shoulder are usually identifiable through the use of plain radiographs in an emergency department. However, other significant soft tissue injuries can be missed at initial presentation. This study used contrast enhanced magnetic resonance arthrography (MRA) to determine the pattern of underlying soft tissue injuries in patients with traumatic shoulder injury, loss of active range of motion, and normal plain radiography.

**Methods:**

A prospective, observational cohort study. Twenty-six patients with acute shoulder trauma and no identifiable radiograph abnormality were screened for inclusion. Those unable to actively abduction their affected arm to 90° at initial presentation and at two week’s clinical review were consented for MRA.

**Results:**

Twenty patients (Mean age 44 years, 4 females) proceeded to MRA. One patient had no abnormality, three patients showed minimal pathology. Four patients had an isolated bony/labral injury. Eight patients had injuries isolated to the rotator cuff. Four patients had a combination of bony and rotator cuff injury. Four patients were referred to a specialist shoulder surgeon following MRA and underwent surgery.

**Conclusions:**

Significant soft tissue pathology was common in our cohort of patients with acute shoulder trauma, despite the reassurance of normal plain radiography. These patients were unable to actively abduct to 90° both at initial presentation and at two week’s post injury review. A more aggressive management and diagnostic strategy may identify those in need of early operative intervention and provide robust rehabilitation programmes.

## Background

Shoulder pain is a significant cause of morbidity in the general population. It is estimated to be the third most common cause of musculoskeletal consultation in primary care even though only approximately 50% of people with shoulder pain will actually consult their family doctors [1]. The majority of shoulder problems have been allocated into three major categories: namely, arthritis, soft tissue injury and intra-articular injury or instability. It is the latter two categories in particular that present diagnostic problems and challenges in emergency medicine and are the focus of this study. The majority of patients seen in the emergency department (ED) following shoulder trauma are investigated using plain radiographs. In the UK those without evidence of fracture or dislocation are typically given the non-specific diagnosis of a ‘shoulder strain’. The nature of this ‘strain’ is usually ill defined, despite the likelihood that soft tissue injuries such as rotator cuff tears, glenoid labrum tears or subacromial bursitis can lead to significant morbidity and some may warrant early surgical intervention.

Ultrasonography (US) and magnetic resonance imaging (MRI) are regularly used to detect shoulder soft tissue injury. In terms of deciding the superior diagnostic test for this injury, a Cochrane review concluded that although either imaging technique could be used for full thickness tears, there were too few studies available to state superiority for partial tears [2]. For other injuries such a glenoid labrum tears, an MRI can be enhanced by an intra-articular injection of radio-opaque dye resulting in magnetic resonance arthrography (MRA). The radio-opaque dye acts as contrast material that helps to delineate intra-articular structures and outline abnormalities.

There are no studies on the examination and imaging diagnosis of *acute* soft tissue shoulder injuries. Systematic reviews [1, 3–7] do not refer to examination in the acute clinical setting and one review excluded all studies which had traumatic origin [1].

We undertook this work as a pilot study in order to ascertain the implications of MRA on acute traumatic shoulder injuries presenting to the ED. The aim was to investigate the impact of MRA on diagnosis and subsequent management and to determine the pattern of underlying soft tissue injuries in patients with traumatic shoulder injury.

## Methods

A prospective observational cohort study was conducted in the ED at Manchester Royal Infirmary. This department is a University affiliated teaching hospital and sees an average of 100,000 acute attendances per year. Clinicians assess over 900 shoulder injuries per year, 67% of which are soft issue injuries. This constitutes the fourth most common musculoskeletal injury in our ED.

### Subjects

Adult patients were consecutively recruited from November 2010 to March 2011. Therefore this was a snap shot of the patients seen at the ED over a period of time for which funding for the gadolinium enhanced MRI scans was available. Patients presented to the ED with a significant, acute, traumatic injury to the shoulder within the previous 48 h. All underwent physical examination by an ED clinician involved in the project (MJC, JPB, DH, SC,) who requested plain radiographs. Inclusion and exclusion criteria were then applied based on the clinical and radiographic results.

### Inclusion criteria

Patients were included if there was no evidence of fracture or dislocation on plain radiography and were unable to actively abduct the shoulder beyond 90°. This joint angle was chosen based on the opinion of the study research team as an easy shoulder angle to assess accurately in clinical practice without a goniometer. Patients with these criteria were reviewed two week’s later in the ED. At this review, if patients were symptomatic and still unable to actively abduct their shoulder beyond 90° were invited to participate in the study. Those who could abduct their shoulder beyond 90° after two weeks were discharged to their GP.

### Exclusion criteria

Patients were excluded if they had a clear evidence of bone or joint injury on plain radiography or if they were pregnant, in prison custody, had previous ipsilateral shoulder surgery and previous ipsilateral documented shoulder pathology or were unable to provide informed consent. For the MRA, additional exclusions were claustrophobia, cochlear implants, any metal objects in the body including joint prostheses, cardiac or neural pacemakers, hydrocephalus shunts, kidney dysfunction with an eGFR ≤44 ml/min or on renal dialysis, intrauterine contraceptive device or a coil.

All patients provided full written informed consent. Ethical approval was granted by the North West Local Research Ethics Committee (10/H1005/12).

### Imaging procedures

Contrast enhanced imaging was performed using a 1.5Tesla scanner (Phillips Gyroscan ACS NT (Phillips, Best, NL) at the NIHR Wellcome Trust Clinical Research Facility, Manchester. Immediately prior to imaging, patients had an intra-articular injection of the glenohumeral joint with 20 mls of 0.5% gadolinium in normal saline. All injections were performed under ultrasound guidance by specialist emergency clinicians. The MRA sequences taken were axial, sagittal and coronal obliques with T1 fat saturated (FS) imaging, proton density FS coronal oblique volume of the shoulder and Short Tau Inversion Recovery (STIR) coronal oblique of the shoulder. These were scored for injury to bone and to all soft tissue structures including the glenoid labrum. All images were reviewed and scored by the study radiologist (CEH) who had 25 years of experience in musculoskeletal imaging and who was blinded to all other clinical data.

### Outcome measures

The principle outcome measure for this pilot study was the presence and diagnosis of an identifiable injury, as determined by MRA. The specific lesions were lesions to any component of the rotator cuff, lesions to the glenoid labrum, the subacromial bursa, the capsule, the acromioclavicular joint and bony lesions such as bone marrow edema and fracture.

### Statistical analysis

Descriptive statistics only were calculated for this study. No formal sample size calculation was performed due to the feasibility nature of the project. Level of statistical significance was not required.

## Results

A total of 26 patients were recruited during the study period. Their mean age was 44 years (Range 21–80) and there were four females.

**Fig. 1:**
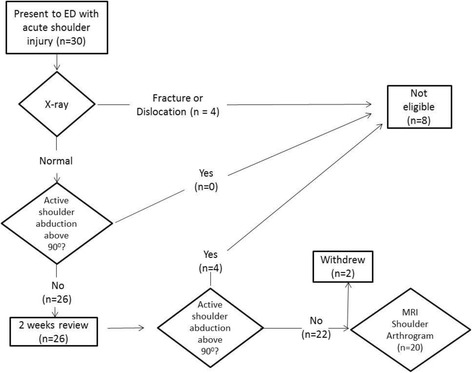
study process flowchart

Four patients who were unable to abduct their shoulder to 90° at first presentation had full active movement at the 2 week’s review clinic appointment. These patients were not included in the study and did not undergo MRA as the examining clinician was satisfied that they were recovering symptomatically and functionally. Of the remaining 22 eligible patients, one patient consented to the study but body habitus precluded MRA. Another patient initially agreed to take part in the study but withdrew on the day of the scan. Thus 20 patients were eventually scanned see Fig. 1; their characteristics and MRA diagnoses are shown in Table 1.

**Table 1:** Patient characteristics at time of presentation and MRA findings

Number	Side	Mechanism of injury	MRA findings
1	L	Lifting heavy weight felt snap	Full thickness tear of SST and SScapT
2	R	Fall down steps	Bruising to deltoid
3	R	Lifting heavy object felt snap	Possible Posterior labral tear
4	R	Jerk to shoulder felt whilst caring for & lifting wife	Full thickness tear of SST and partial tear of SScapT tendon
5	L	Fall onto shoulder whilst snowboarding	Normal images
6	L	Fall onto arm playing football	SLAP lesion with undisplaced humeral neck fracture
7	L	Dived into swimming pool	Evidence of anatomically resolved dislocation. Stripping of anterior capsule from glenoid.
8	L	Slipped down embankment with shoulder abducted	IFST tear SScapT tear
9	L	Alleged assault to shoulder	Posterior capsule tear and posterior superior labral tear
10	R	Rugby. Fall in abduction onto shoulder	Middle glenohumeral tear and partial SST tear
11	L	Fall plus direct blow to shoulder against wall	Torn SST tendon, torn SScapT, subluxed biceps tendon. Capsular tear.
12	R	Trip and Fall with direct trauma to shoulder	Partial SST tear and degenerative ACJ
13	L	Arm pulled backwards into excessive abduction.	Tear of distal aspect SST. Probable fracture greater tuberosity.
14	L	Fall whilst ice skating	Partial tear of the distal aspect of the SST Fracture of the greater tuberosity
15	L	Fall from low roof	Minor ACJ disruption and a possible bone bruise/undisplaced fracture of the clavicle
16	R	Roof fell onto shoulder	Tear of the SST musculo-tendinous junction and a bone bruise of the outer clavicle
17	L	Hit with Lacrosse stick	Full thickness tear of the SST.
18	R	Alleged assault	Appearances suggest minor ACJ injury, though no bone bruise or fracture seen.
19	R	Pulled side tarpaulin on truck	SST tear
20	R	tripped on pavement direct trauma to shoulder	Full thickness tear of the SST, a partial thickness tear of the SScapT tendon and a subluxed biceps tendon.

Abbreviations: *SST* Supraspinatus Tendon, *ACJ* Acromio-clavicular Joint, *SScapT* Subscapularis Tendon, *SLAP* Superior Labral Anterior Posterior, *IFST* Infraspinatus Tendon

The clinical findings at initial presentation and at the review two weeks later are in Table 2. There were no adverse events during the scanning procedures. There were no a priori criteria for patients to have surgical repair. All patients with abnormal MRA were referred to a specialist shoulder surgeon for a further clinical review. Three of these eventually underwent a shoulder surgical procedure. Patient 1 had a hydrodilatation, patient 7 had a Bankart repair, and patient 8 had an arthroscopic labral repair and sub-acromial decompression.

**Table 2:** Details of clinical examination at presentation to ED and at 2 weeks follow up

Patient number	Initial presentation clinical findings	Clinical findings at 2 weeks follow up
1	Active Abd 45; active flexion 90; positive Speed’s test, empty can test. SSS = 9/12	Active Abd 45; Active Flexion 90; positive Neer test, Empty can test Speed’s test, O’Brien’s test; SSS = 8/12
2	Active Abd 145; active flexion 150; positive Speed’s test, empty can test, resisted Ext Rot, Lift off test. SSS = 11/12	Not available
3	Active Abd 20; active flexion 45; unable to do any shoulder specific tests. SSS = 11/12	Active Abd 160; Active Flexion 170; positive Neer test; empty can test; SSS = 11/12
4	Active Abd120; Active flexion120; positive empty can test; SSS = 7/12	Active Abd 170; Active Flexion 170; positive Speed’s test; O’Brien’s test; SSS = 5/12
5	Active Abd 80; Active Flexion100; positive painful arc, empty can test, O’Brien’s test; SSS = 5/12	Active Abd 90; Active Flexion 90;positive Neer test; Hawkins test; O’Brien’s test; SSS = 2/12
6	Active Abd 90; Active Flexion 130; positive painful arc; empty can; lift off test. SSS = 8/12	Active Abd 120; Active Flexion 100 positive painful arc; Neer test; Hawkins test; empty can test; O’Brien’s test; SSS = 10/12
7	Active Abd 180; Active Flexion 180; positive lift off text, crank test, O’Brien’s test. SSS = 6/12	Active Abd 180; Active Flexion 180 positive painful arc; lift off test; empty can test; Neer test; crank test, O’Brien’s test. SSS = 6/12
8	Active Abd 90; 100; Positive Neer test; Hawkins test; empty can test; Speed’s test; O’Brien’s test. SSS = 8/12	Active Abd 80; Active Flexion 150 positive empty can, resisted Ext Rot; Speed’s test SSS = 7/12
9	Active Abd 50; Active flex 60; positive empty can test. SSS = 7/12	Active Abd 50; Active Flexion 45; Neer test; Hawkins test; empty can test; O’Brien’s test; resisted Ext Rot. SSS = 7/12
10	Active Abd 70; Active Flex 80; positive painful arc; Neer test; resisted Ext Rot; Crank test. SSS = n/a	Not Available
11	Active Abd 30; Active Flexion 30; positive Neer test. SSS = 12/12	Active Abd 45; active flexion 20; positive empty can test, O’Brien’s test. SSS = 11/12
12	Trip and Fall onto shoulder Active Abd 20; Active Flexion 40; positive painful arc; SSS = 12/12	Active Abd 50/ Active flexion 90; positive painful arc, empty can test; SSS = 7/12
13	Active Abd 120; Active Flexion 120; positive painful arc, Neer test; Hawkins test; O’Brien’s test; SSS = 9/12	Active Abd 120; Active Flexion 120; positive painful arc, Neer test; Hawkins test; O’Brien’s test; SSS = 9/12
14	Active Abd 30; Active Flexion 30; positive resisted Ext Rot. SSS = 10/12	Active Abd 25 Active Flexion 25; positive Neer test Hawkins; resisted Ext Rot; SSS = 9/12
15	Active Abd 60; Active Flexion 60; positive painful arc; empty can test; SSS = 9/12	Not Available
16	Active Abd 70; Active Flexion 80; positive painful arc; Resisted Ext Rot; SSS = 9/12	Active Abd 40; Active flexion 45; unable to do any shoulder specific tests.SSS = 9/12
17	Active Abd;??Active Flexion??; positive Speed’s test; SSS = 10/12	Active Abd 145;Active Flex 120; positive Neer’s, Hawkins, empty can, Lift off test; O’Brien’s test
18	Active Abd 80; Active Flexion 80; unable to do any shoulder specific tests. SSS = 12/12	Active Abd 45; Active Flexion 80; painful arc;positive Neer; Hawkins. SSS = 12/12
19	Active Abd 90; Active Flexion 80; painful arc, Neer test; Hawkins test; positive empty can; Speed’s test SSS = 11/12	Active Abd 100; Active Flexion 90; painful arc; positive Neer; Hawkins’empty can; O’Brien’s; SSS = 10/12
20	Tripped on pavement and fell onto shoulder Active Abd 70; Active Flexion 90; painful arc; positive empty can test; SSS = 7/12	Active Abd 180; Active Flexion 180; positive empty can; SSS = 9/12

Abbreviations: *Abd* Adbuction, *SSS* shoulder specific score, *Ext Rot* External rotation of the shoulder

## Discussion

The findings in this pilot study are twofold. Firstly, as described in Table 1, there is a high incidence of significant soft tissue and bony pathology in patients presenting to an ED with acute shoulder injury who have normal plain radiographs. The significant injury may be identified by being unable to abduct to 90° at initial presentation in the ED with persistent significant active movement limitation at two week’s review. Secondly, four of these patients had injuries which needed surgical intervention. As such, the standard ED practice of discharging patients to primary care after an acute shoulder injury with a significant loss of active range of motion and a normal plain radiograph should be questioned.

This study was designed as an exploratory project to assess the potential impact of early investigation of shoulder injuries. We believe that our findings warrant further investigation. It is clear that requesting only plain film assessment for significant shoulder injury with very limited active abduction will miss a number of clinically important lesions. This represented the vast majority of patients in our cohort. This may be a result of imposing the relatively stringent definition of severe soft tissue injury as being unable to actively abduct to 90° at initial assessment and at 2 weeks. This particular inclusion criterion was based on the opinion of the study research team as an easy shoulder angle to assess accurately in clinical practice without a goniometer. The early diagnosis of a structural injury may be an indicator of patient outcome, but we were unable to conduct sufficiently long term follow up to determine whether spontaneous resolution of function occurred.

The results of the MRA were shared with participants and clinicians. This led to orthopaedic referral and physiotherapy intervention. Although we believe it likely that early referral and treatment would benefit patients compared to a delayed referral, this question cannot be addressed in this trial design.

**Fig. 2:**
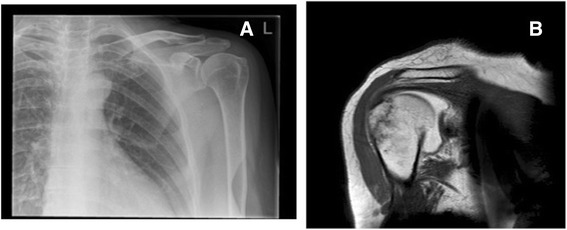
**a** Left shoulder Plain radiography coronal plane which was reported as normal and (**b**) the subsequent MRA on the same patient (Left shoulder) which revealed an occult fracture of the greater tuberosity

**Fig. 3:**
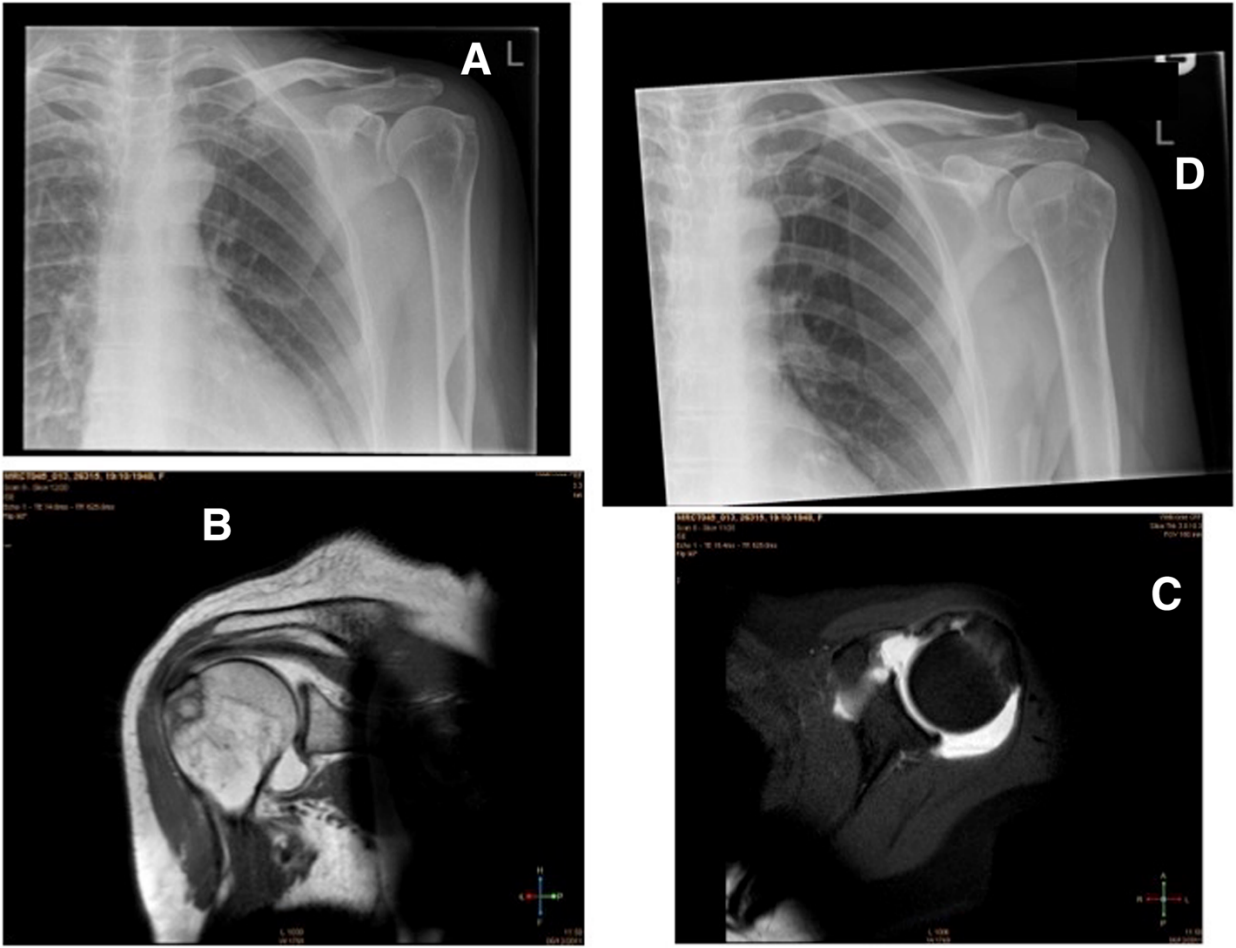
**a** Left shoulder Plain radiograph coronal plane reported as ‘normal’. **b** Coronal plain MR scan and (**c**) MR arthrogram both showing occult fracture greater tuberosity. **d** Plain radiograph coronal plain X-ray taken 2 weeks later showing undisplaced fracture greater tuberosity

**Fig. 4:**
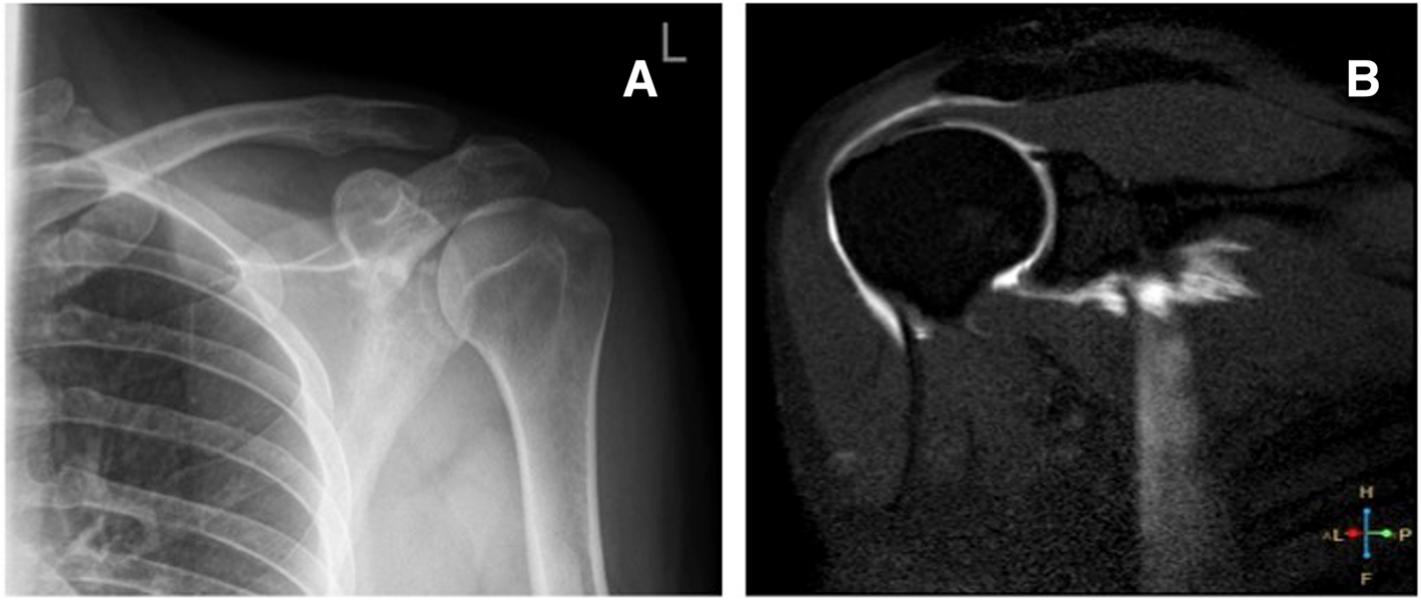
**a** Plain radiograph in coronal plane left shoulder. No acute abnormality reported. Incidental calcific deposit noted. **b** MR arthrogram coronal plane of same patient showing tear of subscapularis tendon and leakage of contrast agent

There are surprisingly little data in the literature on the role of advanced imaging techniques of the shoulder in the Emergency Department. As such, comparison with previous literature is difficult. This is the first attempt at early diagnosis with MRA of patients after acute shoulder trauma. Therefore the importance of diagnoses is important so that the patient can be appropriately referred for surgical or non-surgical management. This is emphasised by only one individual in our study having a normal MRA (Table 1 no. 5). One surprising finding was that four patients had fractures which were not visible on the plain film examination (e.g Fig. 2a and b; Fig. 3a, b, c and d). This highlights the usefulness of MR imaging to correctly diagnose these occult injuries and ensure the proper advice was given to patients regarding either mobilisation or rest. Nevertheless, we would urge some caution amongst Emergency Department clinicians who interpret our findings as suggesting all those who present with shoulder injuries should have an MRA. Our study’s aim was to use MRA to determine the pattern of underlying soft tissue injuries in patients with traumatic shoulder injury but who had a normal plain radiograph (e.g. Fig. 4a and b). The nature of our study also enabled us to see how MRA could be part of a decision making process. It is important to note that this does not exclude other decision making routes such as the effect on pain and impingement of a subacromial injection of local anaesthetic.

It might be argued that current ED management of shoulder acute injury is analogous to the knee joint twenty years ago. Historically patients with significant non-bony knee trauma such as anterior cruciate ligament ruptures were diagnosed in ED as ‘knee sprains’ and only investigated further if they had persistent problems, regular re-injury and re-presentation to ED. Currently, shoulder injuries follow a similar pathway. We believe that this study highlights the need to take these injuries in the shoulder more seriously and to investigate further whether early correct diagnosis, assessment and intervention improves patient outcome.

## Conclusion

There is a high incidence of significant shoulder soft tissue injury definable on MRA amongst ED patients after trauma and an inability to actively abduct to 90° at initial presentation and two weeks later. Further work is required to determine whether early intervention will improve functional outcome in such patients.
